# A comparative biology approach to DNN modeling of vision: A focus on differences, not similarities

**DOI:** 10.1167/jov.21.10.17

**Published:** 2021-09-22

**Authors:** Ben Lonnqvist, Alban Bornet, Adrien Doerig, Michael H. Herzog

**Affiliations:** 1 Laboratory of Psychophysics, Brain Mind Institute, École Polytechnique Fédérale de Lausanne (EPFL), Lausanne, Switzerland; 2 Laboratory of Psychophysics, Brain Mind Institute, École Polytechnique Fédérale de Lausanne (EPFL), Lausanne, Switzerland; 3 Donders Institute for Brain, Cognition and Behaviour, Nijmegen, Netherlands; 4 Laboratory of Psychophysics, Brain Mind Institute, École Polytechnique Fédérale de Lausanne (EPFL), Lausanne, Switzerland

**Keywords:** deep neural networks, modeling, comparative biology, crowding, illusions

## Abstract

Deep neural networks (DNNs) have revolutionized computer science and are now widely used for neuroscientific research. A hot debate has ensued about the usefulness of DNNs as neuroscientific models of the human visual system; the debate centers on to what extent certain shortcomings of DNNs are real failures and to what extent they are redeemable. Here, we argue that the main problem is that we often do not understand which human functions need to be modeled and, thus, what counts as a falsification. Hence, not only is there a problem on the DNN side, but there is also one on the brain side (i.e., with the explanandum—the thing to be explained). For example, should DNNs reproduce illusions? We posit that we can make better use of DNNs by adopting an approach of comparative biology by focusing on the differences, rather than the similarities, between DNNs and humans to improve our understanding of visual information processing in general.

## The explanans

Deep neural networks (DNNs) have revolutionized the field of computer vision, reaching or exceeding human performance in object recognition tasks (LeCun, Bottou, Bengio, & Haffner, 1998; Krizhevsky, Sutskever, & Hinton, 2012; Simonyan & Zisserman, 2015; He, Zhang, Ren, & Sun, 2015). This excellent performance and the analogy between DNNs and the primate visual cortex have caused a fierce discussion about the use of DNNs as neuroscientific models of the brain (e.g., Rosenholtz, 2017; VanRullen, 2017; Majaj & Pelli, 2018; Kubilius, 2018; Cichy & Kaiser, 2019; Kietzmann, McClure, & Kriegeskorte, 2019a; Richards et al., 2019; Firestone, 2020; Griffiths, 2020; Lindsay, 2020; Saxe, Nelli, & Summerfield, 2020; see also DiCarlo, Zoccolan, & Rust, 2012). This debate is about the explanans—that is, how good DNNs are as *models* for human brain processing and behavior.

It is first important to make a distinction among the different types of models (Cichy & Kaiser, 2019). We distinguish three general groups: functional models, which attempt to capture the most important behavioral characteristics of a system; mechanistic models, which attempt to recreate the underlying implementation of the system itself; and replica models, which attempt to do both as accurately as possible. For example, a replica model of the human brain might be an in silico model, where all details, neural spikes, and mRNA coding, for example, are captured. The Human Brain Project (Markram et al., 2011; Amunts, Ebell, Muller, Telefont, Knoll, & Lippert, 2016) is an example that aims to create such a replica. With such a model, neural processes could be studied as if they were factually human brain processes. For example, one may knock out the dopaminergic system and study what neural functions it contributes to. Whether such models are practically possible is an open question. When we use the term “model,” we do not consider replica models unless mentioned explicitly.

Typically, models attempt to describe only some aspects of a system. These models abstract away certain aspects either by condensing complex processes into simpler components or by ignoring them entirely. A model of a neuron may ignore the complex molecular machinery in the nucleus of the neuron without sacrificing the predictive power of certain aspects. Such a model is functional, as it does not explain (nor does it attempt to) the mechanisms by which the neural responses are generated.

Mechanistic models, as opposed to functional models, attempt to describe the mechanisms by which certain behaviors of the system arise. A mechanistic model of a neuron, unlike a functional one, would not ignore the complex molecular biology underlying the function of the neuron; instead, it would attempt to explain how those processes contribute to the behavior of the neuron but not necessarily the entire system.

The debate about the use of DNNs as models of human vision centers on several arguments, of which the following three are of most importance.

First and foremost, DNNs can predict neural activation in primate visual cortex better than other preceding models (Cadieu et al., 2014; Khaligh-Razavi & Kriegeskorte, 2014; Yamins, Hong, Cadieu, Solomon, Seibert, & DiCarlo, 2014; Kubilius, Schrimpf, Nayebi, Bear, Yamins, & DiCarlo, 2018; but see Eberhardt, Cader, & Serre, 2016; Xu & Vaziri-Pashkam, 2021). This fact is strong evidence that DNNs share something crucial in common with human visual processing that other traditional models do not (whether it be general similarities of architecture, optimization, or something else).

Second, DNNs have shown great promise for modeling human psychophysical tasks, such as image recognition (e.g., Geirhos, Rubisch, Michaelis, Bethge, Wichmann, & Brendel, 2018; Su, Vargas, & Kouichi, 2019; Geirhos, Meding, & Wichmann, 2020; Geirhos, Narayanappa, Mitzkus, Thieringer, Bethge, Wichmann, & Brendel, 2021) or crowding, a breakdown of object recognition in the presence of surrounding objects (Volokitin, Roig, & Poggio, 2017; Doerig, Bornet, Choung, & Herzog, 2020a; Lonnqvist, Clarke, & Chakravarthi, 2020). However, even though DNNs show close to human-like object recognition performance, their processing can be highly different than that of humans. For example, ImageNet-trained DNNs prefer textural information rather than the shape-based information that humans prioritize (Geirhos et al., 2018). The trial-by-trial performance of DNNs in perceptual tasks is also consistently different than that of humans (Geirhos et al., 2020; Geirhos et al., 2021). Likewise, although on a category-to-category basis the response patterns of DNNs may appear similar to those of humans, the specific images on which DNNs make misclassifications are often different from the images on which humans make misclassifications (Geirhos et al., 2020). This suggests systematic differences in categorization ability. Even the specifically brain-inspired recurrent CORnet-S shows response patterns that are similar to those of other DNNs and dissimilar to human response patterns (Rajalingham, Issa, Bashivan, Kar, Schmidt, & DiCarlo, 2018; Geirhos et al., 2020). This indicates that the function they compute to solve a task, regardless of architectural specifics, remains largely different from that of humans. Hence, even though performance of DNNs and humans may be similar, the computation underlying the performance may be very different.

Finally, DNNs are prone to overfitting and adversarial attacks (e.g., Goodfellow, Shlens, & Szegedy, 2014; Su et al., 2019; Dujmović, Malhotra, & Bowers, 2020). An interesting example is the one-pixel attack (Su et al., 2019), whereby changing a single pixel in an image can cause a DNN to misclassify the image. These attacks may depend on the dataset, however, rather than on the model architecture (Ilyas, Santurkar, Tsipras, Engstrom, Tran, & Madry, 2019; Mehrer, Spoerer, Jones, Kriegeskorte, & Kietzmann, 2021). This implies that DNNs trained with vulnerable datasets, such as ImageNet and CIFAR, are not good representatives of the potential of DNNs to serve as models of human vision, as humans are not vulnerable to such adversarial attacks. There is ongoing research as to what defenses can be employed to make DNNs robust to adversarial attacks (for reviews, see Xu, Ma, Liu, Deb, Liu, Tang, & Jain, 2019; Machiraju, Choung, Frossard, & Herzog, 2021), and recent research has demonstrated large improvements in robustness (e.g., Dapello et al., 2020; Radford et al., 2021).

Taken together, there are clear shortcomings of DNNs as models of the human system. The question is how serious these shortcomings are. Firestone (2020) pointed out that human–DNN comparisons may often not be “fair.” Indeed, human–DNN comparisons have little meaning if the methods by which humans and DNNs are tested are dissimilar. Firestone argues that the process for making these comparisons fair is threeway: One must limit DNNs like humans, limit humans like DNNs, and align tasks to consider the species performing them. It remains unclear whether DNNs generally fail to exhibit human-like effects in visual phenomena or whether this dissimilarity is caused by unfair comparisons. As Firestone (2020) argued, suppose a DNN is optimized to exploit image-level textural information due to a bias in a dataset. It would then not be fair to say that, because this DNN places more emphasis on textural information (as opposed to, for example, shape information), DNNs generally process information differently than humans do (Geirhos et al., 2018). Given these conflicting arguments and studies, the status of DNNs as models of information processing in the human visual system remains unclear.

## The explanandum

In the last section, we reviewed the discussion of whether or not DNNs describe human brain processing and behavior well; that is, the focus was on the explanans. In this section, we argue that there is an issue with the explanandum, as well; we do not understand brain processing well enough to determine what is crucial and what is not. Hence, we cannot know what can or should be abstracted away by a model and what cannot or should not be. In other words, we often do not know when a model is falsified and when it is not. If we do not know what phenomenon we want to explain, the question of whether or not DNNs are good at explaining the phenomenon is irrelevant.

### The neural explanandum

Here, we describe cases where we know little about neural processing. First, it is a great success that DNNs can predict neural responses well in terms of correlations of DNN and primate neural spike rates. However, it is still a mystery what the neural code of the brain is, and perhaps it is possible that specific spike rates are less crucial than thought; thus, these correlations may only pick up some epiphenomenal aspects of brain processing. Second, we argue that metrics such as the Brain-Score (Schrimpf, 2018) may not provide sufficient constraints for meaningful model selection on a neural level due to the low resolution of the data on which they are computed.

When modeling the human visual system and judging success rates based on a metric, we rank different explanans (explanations) of the underlying explanandum (in this case, the human visual system) according to this metric. The metric (e.g., Brain-Score; Schrimpf, 2018) serves as a ranking of the explanans for that explanandum. In other words, we are explaining performance on a metric using our models and hoping that improvements in explaining the metric generalize to improvements in explaining the underlying system (the brain).

One problem with this approach is that current metrics do not allow model selection. For example, the Brain-Score benchmark (Schrimpf et al., 2018; Schrimpf, Kubilius, Lee, Ratan Murty, Ajemian, & DiCarlo, 2020) combines a number of neural predictivity scores, such as correlations between neural activity in DNN layers and the visual cortex (V1, V2, V4) and inferior temporal (IT) cortex, as well as correlations between behavioral measures of DNNs and humans. Interestingly, many of the top-ranking models on the Brain-Score are architecturally substantially different, yet show similar performance in terms of Brain-Score. For example, simulating the human primary visual cortex at the front of a convolutional network (Dapello et al., 2020) barely improves Brain-Score compared with other models, such as the early brain-inspired VGG-19 (Simonyan & Zisserman, 2015); a recurrent brain-inspired model, CORnet-S (Kubilius et al., 2018), or other convolutional networks (He et al., 2015; Huang, Liu, Van Der Maaten, & Weinberger, 2017).

This is problematic because these metrics fail to adequately distinguish among substantially different model architectures. Although there are properties of DNNs that generalize to the human visual system according to the Brain-Score metric (such as convolutions), direct modeling efforts have not been able to make substantial progress in explaining the human visual system beyond this. If a metric fails to discriminate among models that are recurrent (Kubilius et al., 2019), fully feedforward (Simonyan & Zisserman, 2015), residual (He et al., 2015; Huang et al., 2017), or other (e.g., Kolesnikov, Zhai, & Beyer, 2019; Dapello et al., 2020), then perhaps the metric is insufficient. The main problem is that model failure is not clearly defined in this case; either the Brain-Score metric or the methodology with which a model is evaluated on it fails to distinguish among what we would think of as fundamentally different types of model architectures.

One possible cause of the problem is the low resolution of neural imaging data. In Brain-Score, for example, correlation in V4 and IT is computed on the activity of 88 V4 neurons and two datasets of 168 IT neurons and on five 96-electrode Utah arrays in IT, respectively (Freeman, Ziemba, Heeger, Simoncelli, & Movshon, 2013; Majaj, Hong, Solomon, & DiCarlo, 2015; Schrimpf et al., 2018; Kar, Kubilius, Schmidt, Issa, & DiCarlo, 2019). In a recent article, Xu and Vaziri-Pashkam (2021) demonstrated (in a functional magnetic resonance imaging study) that higher neural resolution data can allow for substantially better model discrimination. This suggests that more work is needed on collecting higher resolution datasets to allow better model selection based on neural metrics.

Similar results are found in human studies. Predicting the human brain activity of one human with the neural activity of another in different modalities, such as vision (Hasson, Nir, Levy, Fuhrmann, & Malach, 2004) and social interaction (Dumas, Nadel, Soussignan, Martinerie, & Garnero, 2010), is not much more precise than predicting human neural data with DNNs. This has been formalized as a “noise ceiling,” an upper bound on the linear predictability of the human brain derived from predictability of the human brain from other primates, and recent evidence suggests this ceiling may have been reached (Khaligh-Razavi & Kriegeskorte, 2014; Storrs, Khaligh-Razavi, & Kriegeskorte, 2020a; Storrs, Kietzmann, Walther, Mehrer, & Kriegeskorte, 2020b; but see Xu & Vaziri-Pashkam, 2021).

Current metrics such as the Brain-Score are not sufficient to understand what is relevant for the human visual system, as they mask important aspects about how a task can be performed in different ways. In this case, several architectures can perform similarly along the Brain-Score metric, and hence it is unclear what architectural parts of DNNs are crucial in this respect. For this reason, it remains unclear which neural code is used by the visual cortex.

### The psychophysical explanandum

Just as it is difficult to find which neural activities to model, deciding which behavioral characteristics to model is not straightforward, either. Here, we discuss the importance of being explicit about the phenomena we wish to model and about which results would falsify a model.

Scientists may have reasons to think some phenomena of human vision are idiosyncratic, but others are ubiquitous and crucial for understanding visual processing. Studying seemingly idiosyncratic phenomena using traditional models often requires careful thought about how to phrase the phenomenon in a way that allows for the model to clearly display these effects. In contrast, DNNs allow us to study either type of phenomenon with relative ease compared with traditional types of models. This is because model formulation itself may involve as little as selecting an existing DNN architecture, and a dataset to train it with. Whether or not the underlying phenomenon being modeled is ubiquitous is not important from the point of view of the model. DNNs allow us to conveniently place the explanans before the explanandum because they are not generally explicitly hypothesis driven (barring superficial architectural similarities). In other words, justifying why a phenomenon (such as a visual illusion) should be modeled under the DNN framework is easily ignored.

It is therefore doubly important that we carefully justify the phenomena we model using DNNs to avoid finding patterns where there are none. In other words, let us not “blanket model” every phenomenon simply because we can.

Here, we show that it is not always clear which phenomena should be modeled and which should not. We illustrate this with two phenomena: crowding and illusions. We argue that, in the case of visual crowding, we know what we want to model and how we can validate and falsify models, but that the same cannot be said for visual illusions.

#### Example 1: Crowding

Crowding is the ubiquitous breakdown of object recognition in the presence of nearby flankers (Bouma, 1970; Pelli, Palomares, & Majaj, 2004; Levi, 2008; Sayim, Westheimer, & Herzog, 2008; Manassi & Whitney, 2018). In simple stimulus configurations, crowding is easily described by Bouma's law (Bouma, 1970; Pelli & Tillman, 2008): Only flankers within a certain window around the target deteriorate performance. The window size is often estimated to be half of the eccentricity of the target location; however, recent work has shown that more complex computational processes underlie crowding. Importantly, when adding flankers, object recognition improves under certain conditions (Manassi, Sayim, & Herzog, 2013; Manassi, Hermens, Francis, & Herzog, 2015; Herzog & Manassi, 2015). This phenomenon, called uncrowding, can occur even when adding flankers outside of Bouma's window. Uncrowding challenges most models of vision because more flankers can only deteriorate performance in these models. For example, a crucial operation in the early layers of DNNs is pooling information across neighboring spatial locations. More flankers diminish target signals and hence psychophysical performance.

Crowding and, relatedly, uncrowding are ubiquitous phenomena in vision, as stimuli are rarely encountered in isolation. In this respect, we argue that any successful object recognition system must cope with crowding because of its ubiquity; if a model does not produce crowding and uncrowding, it should be rejected. Study of such models could provide insight into the purpose and consequences these phenomena may carry for visual processing systems (e.g., see Doerig, Schmittwilken, Sayim, Manassi, & Herzog, 2020b, who show evidence for the importance of recurrent segmentation in visual processing).

On the other hand, crowding shows interesting phenomena such as anisotropies (Toet & Levi, 1992), whereby flankers away from fixation crowd more strongly than flankers closer to fixation. We would not require a model to explain this phenomenon, as we have in the model-building process abstracted away from heterogeneities in the photoreceptor distribution, which are believed to cause these anisotropies. Hence, crowding is a function of the visual system where we likely know what counts as a failure of a model in the context of vision and what does not. The rejection criteria for models are clear, because we likely know what we *should* model and are explicit about what it is we are *attempting* to model.

Many DNNs have failed to reproduce uncrowding, highlighting that they process visual information very differently from humans (Doerig et al., 2020a; Lonnqvist et al., 2020). However, adding an explicit segmentation stage can remedy the models (Doerig et al., 2020b). Thus, DNNs are not rejected as models of human vision in general, but only certain types of DNNs are. In this case, we have successfully done model selection.

#### Example 2: Illusions

Here, we argue that visual illusions are a case where the explanandum is not clear, and because of that modeling visual illusions may be premature. We do not currently understand in most cases why illusions appear in the human visual system; for example, it is not known whether they are “bugs” of the visual system or whether they are a feature (for a discussion about veridicality of illusions, see, e.g., Braddick, 1972; Braddick, 2018; Todorović, 2018; Todorović, 2020). One may argue that DNNs need to capture visual illusions simply because they are part of human vision, but this can be said about all visual function and we return to the replica model case. Alternatively, one may argue that illusions are just idiosyncratic failures of the visual system that we should abstract away as in the case of the anisotropies of crowding.

Many studies have investigated whether and to what extent DNNs are susceptible to illusions, and they have found an array of conflicting results. For example, training DNNs on complex tasks made them susceptible to visual illusions (Mély, Linsley, & Serre, 2018; Watanabe, Kitaoka, Sakamoto, Yasugi, & Tanaka, 2018; Benjamin, Qiu, Zhang, Kording, & Stocker, 2019; Gomez-Villa, Martín, Vazquez-Corral, & Bertalmío, 2019), but not consistently so (Gomez-Villa, Martín, Vazquez-Corral, Bertalmío, & Malo, 2020). Gomez-Villa et al. (2020) showed that small DNNs do not exhibit the gradient illusion like humans but exhibit many classic illusions (such as the dungeon illusion and White's illusion). In contrast, state-of-the-art DNNs (Zhang, Zuo, Chen, Meng, & Zhang, 2017; Tao, Gao, Shen, Wang, & Jia, 2018) exhibit human-like effects in the gradient illusion but weak to no effect on others. In general, it appears difficult to find a coherent way of relating these conflicting results to human illusory processing (e.g., Baker, Erlikhman, Kellman, & Lu, 2018; Sun & Dekel, 2019; Ward, 2019). Thus, should we dismiss all models because none of them reproduces all human illusions, or are some illusions more important than others, allowing us to do model selection accordingly? We simply do not know. The problem is with the explanandum, not with the explanans.

In fact, the situation is even more complex. The lack of coherence is not limited to DNNs but also extends to human studies. Humans exhibit large individual variation in illusion strength, and performance in most illusion tasks poorly predicts performance in other illusions, even when the illusions are qualitatively similar (Grzeczkowski, Clarke, Francis, Mast, & Herzog, 2017; Cretenoud, Karimpur, Grzeczkowski, Francis, Hamburger, & Herzog, 2019; Cretenoud, Grzeczkowski, Bertamini, & Herzog, 2020). Importantly, some humans are not deceived by certain illusions at all. Thus, what should we model? The problem is not the heterogeneity of visual illusions per se (this issue can be escaped by modeling specific illusions on a case-by-case basis) but rather the fact that, because of the large degree of heterogeneity in visual illusions even within subjects, it is not yet possible to determine which illusions are crucial for understanding vision. This raises the question of which illusions need to be modeled in a successful model. There is no good answer that we know of.

The fact that there is little coherence within DNN studies and, similarly, little coherence in human studies should not be taken as evidence of the similarity of DNNs to humans in illusory perception. The real problem relates to the human brain and about how it is impossible to know which illusions (if any) are important to human vision. In summary, until we know which illusions reflect crucial and ubiquitous aspects of human visual processing (as we argued that we do in the case of visual crowding), attempting to model them in a vacuum does us little good. We are unable to place our findings into context and will find difficulty in discriminating between a successful and an unsuccessful model. It may be that the situation is similar for other visual functions, so that it is not clear what needs to be modeled and what aspects should be abstracted away.

## When the explanandum becomes the explanans: An approach of comparative biology

In this section, we argue that we can learn a great deal from DNNs when we consider them as independent visual species—in other words, by considering them as visual systems in their own right rather than as models of the human visual system. The approach is akin to comparative biology research. Here, we offer some important insights we have gained from DNNs under this framework.

First, potentially the deepest insight for the vision community may have been that object recognition can occur without carefully modeling the visual system step by step along its hierarchy. Over the last half century, vision research has been split up in many subcommunities that are concerned with a variety of topics, such as shape, motion, color, and others. Even though there has been little cross-talk between these subcommunities, all of these fields unify the implicit notion that the visual system must be able to solve all of these aspects individually for successful object recognition. DNNs have demonstrated that a system can perform well on object recognition without explicitly training on or even performing well in many of the aforementioned areas (for example, DNNs apparently solve vision without relying on shape information; Geirhos et al., 2018). This provokes the question as to what extent some of the visual processes we have studied in the last 50 years are crucial and representative of vision in general.

Second, a particular question was whether object recognition necessarily requires explicit object segmentation (Herzog, Sayim, Chicherov, & Manassi, 2015) or can occur without it (VanRullen & Thorpe, 2002). DNNs have provided a clear answer to this question in favor of the latter hypothesis (e.g., Krizhevsky et al., 2012; Simonyan & Zisserman, 2015). Here, a new question arises. Does a computational advantage exist in favor of segmentation, or is it simply a suboptimality caused by either our environment or evolution? In this respect, the failure of DNNs to exhibit human-like crowding and uncrowding is not actually a failure but rather a useful clue that may offer insights for what is crucial for which types of visual function.

Third, neuroscience often relies on a subpart coding strategy. The coding of neurons can be mapped directly onto perceptual aspects such as V1 activity to edge perception, IT activity to faces, and even mapping individual faces to neurons. Neurons of DNNs show a coding with relatively interpretable features but not ones reliably mappable to the brain (e.g., Xu & Vaziri-Pashkam, 2021). In addition, different DNNs code subparts differently (e.g., Olah, Mordvintsev, & Schubert, 2017; Geirhos et al., 2018). These aspects are not surprising, as it is known that one can use infinitely many orthonormal bases, such as Fourier or Gabor wavelets, to code for any function (i.e., representation of a stimulus). Hence, there are many ways to code subparts, and DNNs may show the extent to which details of how subparts are coded does not matter. Likewise, artificial vision can be achieved in many different ways, including a large variety of feedforward DNNs (Krizhevsky et al., 2012) or recurrent DNNs (Kietzmann, Spoerer, Sörensen, Cichy, Hauk, & Kriegeskorte, 2019b), with (e.g., Dosovitskiy et al., 2020; Radford et al., 2021) or without (e.g., Krizhevsky et al., 2012) attention and even with very different architectures, such as transformers (Dosovitskiy et al., 2020) and multilayer perceptrons (Tolstikhin et al., 2021).

Finally, DNNs often overfit and show little generalization, as revealed by, for example, adversarial examples (Goodfellow et al., 2014; Ilyas et al., 2019; Su et al., 2019; Dujmović et al., 2020; Mehrer et al., 2021). One may consider this a failure of DNNs. However, we see this fact rather as an invitation to study the question to what extent overfitting is a bug or a feature, given the large resources DNNs and human brains have (Ilyas et al., 2019). In addition, we can ask to what extent humans overfit, as well (for a study about human overfitting, see, e.g., Dubey, Agrawal, Pathak, Griffiths, & Efros, 2018). For example, human perceptual learning is very specific and can be argued to be a case of overfitting (e.g., Spang, Grimsen, Herzog, & Fahle, 2010).

## Conclusions

DNN model selection is currently difficult. We have highlighted issues with regard to this on both the side of the explanandum and the side of the explanans. These issues are not necessarily ubiquitous but can potentially be overcome with further improvement of model validation methods, an increased understanding of different phenomena in the human visual system, and specificity about which phenomena are to be modeled. We argue that, as a whole, accepting DNNs as validated models of the human visual system is premature. In addition, more work is needed to understand what we want to model and what we do not; we should understand the explanandum first. As shown, successful object recognition per se is not a benchmark because it can be achieved in many ways. More fine-grained benchmarks are needed when it comes to both neural processing and psychophysics, and often it is unclear which benchmarks should be used, because we simply do not understand vision in many respects.

Until then, comparisons may be more fruitful when focusing on differences between DNNs and humans. Studying how and why it is possible to achieve a goal differently can offer insight on what is crucial for performing the task. A comparative biology approach can be used as a key step not only in understanding how DNNs function but also in understanding how visual information processing functions *in general* beyond specific species, be it humans, DNNs, or other animals such as chickens (Ciandetti & Vallortigara, 2018). We suggest that we should consider both DNNs and human vision as different subsets of visual information processing; they are different species.

This approach has told us much already; for example, we have learned that large-scale object recognition can be achieved with or without several functions or functional properties of the human visual system, such as attention, segmentation, or recurrence. Ultimately, we think that there can exist a compelling synergy between DNN modeling and neuroscience. A feedback loop of new insights gained from direct modeling (such as new hypotheses or compelling models of specific processes) can be combined with human studies and human–DNN comparative studies to produce a rigorous body of research that facilitates an understanding of the principles underlying visual information processing in general.
